# Contextual determinates of under-five malnutrition in mining communities: a critical review of nutrition interventions for optimal impact

**DOI:** 10.3389/fpubh.2025.1482200

**Published:** 2025-02-12

**Authors:** Herbert Tato Nyirenda, David Mulenga, Hilda Nyambe-Silavwe

**Affiliations:** Department of Public Health, School of Medicine, The Copperbelt University, Ndola, Zambia

**Keywords:** contextual drivers, sustained impact, underweight, under-five children, nutrition intervention, mining communities

## Abstract

**Purpose:**

Malnutrition continues to be a widespread and critical public health issue, yet there is a lack of comprehensive evidence synthesizing empirical findings and assessing the practicality of nutrition interventions in diverse contexts. This paper analyzes contextual data to establish a benchmark for selecting effective nutrition strategies, thereby maximizing their impact and ensuring targeted, sustainable outcomes.

**Methods:**

This study employed a cross-sectional design to examine the key drivers of nutrition in mining communities, focusing on children under five and their caregivers. A sample of 711 participants was selected using a systematic random sampling technique. Data collection involved structured questionnaires, direct measurements of children, and interviews with caregivers. Anthropometric measurements were conducted according to WHO standards to assess underweight status. Statistical analysis included descriptive statistics and chi-square tests to evaluate the effectiveness and feasibility of context-specific nutrition interventions.

**Results:**

Chi-square analysis highlights a complex interplay of factors influencing underweight in children under 5 years of age, including expenditure priorities (*p* = 0.002), access to resources such as primary grocery stores (*p* = 0.001) and farmers’ markets (*p* < 0.001), food preparation practices (*p* = 0.006), agricultural empowerment (*p* < 0.001), and feeding styles/strategies (*p* = 0.004). Multivariate logistic regression further reveals that key determinants of child underweight include age (aOR = 15.24, *p* < 0.001), caregiver disability or chronic illness status (aOR = 0.14, *p* < 0.001), inadequate food production (aOR = 1.94, *p* = 0.009), and expenditure priorities (aOR = 2.46, *p* = 0.007). These factors collectively highlight the multifaceted nature of child undernutrition.

**Conclusion:**

The findings highlight the critical importance of considering contextual factors when developing nutrition interventions. Key elements such as expenditure priorities, access to food resources, food preparation practices, agricultural empowerment, and feeding strategies play a significant role in shaping child nutrition outcomes. Understanding these factors is essential for designing interventions that are not only effective but also sustainable and culturally appropriate.

## Introduction

Malnutrition continues to be a significant global public health issue, impacting individuals of all ages across both developed and developing countries (1). Malnutrition also manifests in various forms, including undernutrition, over-nutrition, and micronutrient deficiencies (2). Children under the age of five (under-five children), particularly those living in vulnerable and resource-poor communities, bear a disproportionately higher burden. Under-five children are particularly susceptible due to their critical growth and development stages, where inadequate nutrition can lead to stunting, wasting, and long-term cognitive and physical impairments. Factors such as limited access to nutritious food, poor healthcare, lack of clean water, and inadequate sanitation compound the risk and the burden of malnutrition (2). Various nutrition interventions, initiatives, and programs have been implemented to address and reduce under-five malnutrition. However, despite these efforts, malnutrition rates remain significantly high which suggests the need to reassess and refine the frameworks for intervention development (3).

Literature on nutrition interventions highlights two key facets of nutrition interventions effectiveness: successful outcomes and areas where conclusions remain inconclusive. On one hand, numerous studies reveal the positive impacts and on the other hand, there is a significant body of literature that points to challenges in achieving consistent results, with many interventions failing to produce sustained improvements or showing limited impact in certain settings (4, 5). The lack of clear conclusions can be attributed to poorly defined intervention strategies, inadequate monitoring and evaluation frameworks, and the failure to consider local contextual factors, such as socioeconomic status, food availability, and cultural preferences (6). Further, evidence indicates that interventions often encounter resistance stemming from differences in conceptualization, functionality, and operationalization (7). To achieve optimal nutrition outcomes through interventions and initiatives, it is crucial not only to prioritize successful implementation but also to utilize context-specific empirical data for tailored approaches (4). To improve the effectiveness of nutrition interventions, it is essential to consider various factors that address the complexity and practicality of the intervention. These include the relative advantage of the intervention, ensuring that it offers clear, tangible benefits over existing practices; trialability, which allows for small-scale testing to evaluate its feasibility and impact before full implementation; and adaptability, which ensures the intervention can be customized to meet the specific needs and conditions of diverse communities (8–11).

Mining communities are shaped by a variety of factors, including the built environment, socio-economic conditions, and cultural norms. Understanding and prioritizing contextual factors creates a foundation for designing interventions that are tailored to local needs, enhancing their effectiveness and increasing the likelihood of long-term, sustained impact. These considerations enable the development of interventions that are not only impactful but also culturally appropriate, scalable, and adaptable to changing needs (12). This paper analyses the contextual drivers of under-five nutrition in mining-communities to provide a benchmark for selecting effective nutrition strategies, thereby optimizing their impact.

## Methods and materials

### Study design and study population

This study employed a cross-sectional study design. The research was conducted in Zambia Sub-Sahara Africa in North-western province, a predominantly rural area, with specific focus on the Solwezi district, encompassing the communities of Kapijimpanga and Kyafukuma where mining serves as the primary economic activity. According to the 2022 population census, Solwezi district had a population of 332,623 with an average annual growth rate of 8.0. The study targeted under-five children and their caregivers. Additionally, this was a baseline evaluation with the aim of understanding the contextual factors surround under-five nutrition to guide intervention development. The aim was to identify context-specific interventions that are both effective and feasible for implementation in mining communities.

### Sample size and sampling technique

Two communities, Kapijimpanga and Kyafukuma, along with their corresponding catchment health facilities (Kapijimpanga and Kyafukuma Health Centre), were selected for the study. A list of all under-five children and their caregivers within the catchment areas of the selected health facilities was compiled, and participants were randomly selected. A sample size estimation formula with a dichotomous outcome was used to estimate a sample of 691 where n_i_ is the sample size required in each group (*i* = 1,2), |p_1_| is the proportions (13% underweight children, 2018 Zambia Demographic Health Survey) under the alternative hypothesis, H_1_, and p is the overall proportion, *α* is the 5% selected level of significance and Z_1-α/2_ is the value from the standard normal distribution holding 1- α/2 below it, and 1- *β* is the selected 80% power and Z_1-β_ is the value from the standard normal distribution holding 1- β below it.

### Data collection

The primary method of gathering data was through a structured questionnaire. A questionnaire was adapted from the Demographic and Health Survey to ensure the validity and reliability. Information was obtained through structured interviews conducted with caregivers, while measurements were directly taken from the children. Various equipment such as measuring boards, measuring tapes, and weight scales were utilized for accurate measurements. A multidisciplinary team consisting of qualified personnel including clinicians, nutritionists, and nurses was responsible for collecting data on nutrition parameters and child health indicators, as well as conducting structured interviews with caregivers. Anthropometric measurements, including age, height/length, and weight, were obtained from the children. To assess anthropometric indices such as underweight, measurements were compared against the WHO Child Growth Standards median by applying the z-score cut-off point of < −2 SD (Standard Deviation) and < −3 SD severe. Malnutrition was assessed using multiple anthropometric indicators, with underweight (measured by weight-for-age) serving as the primary indicator. Therefore, underweight was the dependent variables, offering a holistic view of malnutrition in the population under study.

### Data analysis

The research used a comprehensive statistical analysis methodology that included several key approaches. Descriptive statistics, specifically frequency distributions, were employed to summarize and explore the characteristics of the variables under investigation. Additionally, a bivariate analysis using chi-square was conducted to examine the contextual relationship among individual factors, community-level factors, behavioral factors, and the outcome variable. This analysis aimed to identify potential predictors of underweight. A multivariate logistic regression analysis was conducted to identify and assess the contextual determinants of child underweight, considering a wide range of potential influencing factors. Furthermore, a critical review of effectiveness of nutrition interventions and an analysis of context-driven interventions was performed to offer evidence-based insights and context-specific strategies aimed at optimizing child development and growth interventions.

## Results

### Background characteristics

Table 1 shows findings on the socio-economic and demographic characteristics of respondents. Findings reveal the socio-economic and demographic profile of the respondents. A significant proportion, comprising 74% of the participants, identified themselves as spouses of the household head. Further, the survey showed that 94% of the respondents were female, (54%) were under 25 years old, suggesting a youthful demographic, 85% were married, half (50%) reported having attained a primary school education, an overwhelming majority (95%) of the respondents were not employed and one 6% of the respondents classified themselves as disabled or chronically ill.

**Table 1:** Socio-economic and demographic characteristics of respondent/caregiver.

Characteristics	Percentage %	Confidence interval	Population estimates
Relationship to household head			
Head of the household	19.5	[16.0, 23.5]	138
Spouse	74.2	[69.8, 78.1]	528
Own child	1	[0.4, 2.5]	7
Sister/Brother	1	[0.4, 2.5]	7
Mother/Father	4.3	[2.7, 6.9]	31
Sex of the respondent			
Male	6.5	[4.5, 9.4]	47
Female	93.5	[90.6, 95.5]	665
Age of respondent			
15–19	26.2	[22.2, 30.6]	186
20–24	27.4	[23.4, 31.9]	195
25–29	19.8	[16.2, 23.9]	141
30–34	13.1	[10.3, 16.6]	93
35–39	8.9	[6.5, 12.1]	64
40+	4.5	[2.9, 6.8]	32
Marital status			
Never married	7.8	[5.6, 10.7]	55
Married/living together	85.1	[81.4, 88.1]	605
Divorced	2.3	[1.2, 4.4]	17
Separated	3.6	[2.2, 5.8]	25
Widowed	1.3	[0.6, 2.7]	9
Education level			
None	11.3	[8.7, 14.7]	81
Primary 1–7	50.1	[45.3, 54.8]	356
Basic 8–9	28.1	[24.0, 32.5]	199
High 10–12	10.5	[7.9, 13.9]	75
Employment status			
Informal Employment, i.e., Marketeer, business	2.2	[1.1,4.1]	15
None (Does not work) i.e. housewife	97.8	[95.9,98.9]	696
Disability status			
Disable/Chronically ill	4.6	[3.0,6.8]	32
Able bodied	95.4	[93.2,97.0]	679
Total	100		711

### Status and prevalence of malnutrition in under-five children

Table 2 reveals that 20% of the children surveyed were underweight, with 9% classified as severely underweight. Regarding sex differences, 23% of female children were underweight compared to 19% observed among male children. However, statistical analysis indicated that these differences were not statistically significant (*p* = 0.235). Further, significant variations in underweight prevalence across different age groups were observed (*p* < 0.001). Notably, the prevalence of underweight children increased with age, highlighting a concerning trend as children grow older (Table 3).

**Table 2:** The prevalence of underweight and severe underweight in under-five children.

	%	Confidence interval	Population estimates
Underweight			
None	79.7	[75.6, 83.2]	567
Percentage below −2 SD	20.3	[16.8, 24.4]	144
Total	100		711
Severely underweight			
None	90.6	[87.3, 93.1]	644
Percentage below −3 SD	9.4	[6.9, 12.7]	67
Total	100		711

**Table 3:** Percentage distribution of underweight and child characteristics.

	Underweight			
	None	Percentage below −2 SD	Sample	*p*-value
	%	%		
Sex				
Male	81.3	18.7	352	0.235
Female	76.7	23.3	358	
Total	79	21	711	
Childs age in months				
<6	96.4	3.6	112	<0.001
6–8	95.3	4.7	81	
9–11	88.3	11.7	84	
12–17	71	29	68	
18–23	74.5	25.5	74	
24–35	67.9	32.1	113	
36–47	71.7	28.3	98	
48–59	63.7	36.3	80	
Total	79	21	711	

### Contextual factors associated with under-five nutrition

Table 4 presents the contextual drivers influencing under-five nutrition. The findings reveal compelling correlations between various household factors and the prevalence of underweight children. Households prioritizing expenditures on bills, farm inputs, and other items over food showed higher proportion (37%) of underweight children compared to those prioritizing food expenditure. Access to essential community and public resources also emerged as critical factors. Families lacking access to a main grocery store (25%) or farmers’ market (26%) had more than double the proportion of underweight children compared to those with access to these resources. Furthermore, cooking practices specifically overcooking vegetables was associated with a notable increase in underweight children. Further, Households not engaged farming programs had a 20% higher proportion of underweight children compared to participating households.

**Table 4:** Contextual factors associated with under-five nutrition.

	Underweight					
	None	None	Percentage below −2 SD	Percentage below −2 SD	P-value	Sample
	%	Conf Inter	%	Conf Inter	%	
Expenditure priority						
Food	81.2	[77.0, 84.8]	18.8	[15.2, 23.0]	0.002	625
Bills, school fees, agro-inputs	62.7	[48.9, 74.7]	37.3	[25.3, 51.1]		85
Total	79	[74.9, 82.5]	21	[17.5, 25.1]		711
Access to mainstream grocery store						
Yes	90	[83.1, 94.3]	10	[5.7, 16.9]	0.001	196
No	74.8	[69.8, 79.3]	25.2	[20.7, 30.2]		515
Total	79	[74.9, 82.5]	21	[17.5, 25.1]		711
Access to a farmers market, organic or local food source outlet						
Yes	92.5	[85.6, 96.3]	7.5	[3.7, 14.4]	<0.001	179
No	74.4	[69.5, 78.8]	25.6	[21.2, 30.5]		532
Total	79	[74.9, 82.5]	21	[17.5, 25.1]		711
Time taken cooking vegetables						
Less than 15 min	81.7	[77.4, 85.3]	18.3	[14.7, 22.6]	0.006	601
Less than 30 min	66.6	[50.0, 79.9]	33.4	[20.1, 50.0]		61
Between 30 and 1 h	68.5	[44.3, 85.6]	31.5	[14.4, 55.7]		28
More than 1 h	49.8	[25.1, 74.6]	50.2	[25.4, 74.9]		19
Total	79	[74.9, 82.5]	21	[17.5, 25.1]		711
Trained in farming programmes						
No	78.3	[74.1, 81.9]	21.7	[18.1,25.9]	<0.001	686
Yes	98.9	[92.4, 99.9]	1.1	[0.1,7.6]		25
Total	79	[74.9, 82.5]	21	[17.5,25.1]		711
Feeding strategy-clap hands						
No	75.7	[70.8, 80.0]	24.3	[20.0, 29.2]	0.004	531
Yes	88.8	[81.4, 93.4]	11.2	[6.6, 18.6]		179
Total	79	[74.9, 82.5]	21	[17.5, 25.1]		711
Feeding strategy - make funny faces/play/laugh						
No	76.6	[72.0, 80.7]	23.4	[19.3, 28.0]	0.007	588
Yes	90.5	[81.6, 95.3]	9.5	[4.7, 18.4]		122
Total	79	[74.9, 82.5]	21	[17.5, 25.1]		711
Feeding strategy- modeling how to eat						
No	76.1	[71.3, 80.3]	23.9	[19.7, 28.7]	0.004	556
Yes	89.4	[81.7, 94.1]	10.6	[5.9, 18.3]		154
Total	79	[74.9, 82.5]	21	[17.5, 25.1]		711

### Multivariate logistic regression: relationship between the contextual factors and child underweight

Table 5 presents findings on contextual factors associated with underweight status in children. Age was found to be a significant determinant, with older children being more likely to be underweight, particularly those aged 48–59 months (aOR = 15.24, *p* < 0.001). Other significant factors included the disability or chronic illness status of the caregiver, where able-bodied caregivers were associated with lower odds of underweight (aOR = 0.14, *p* < 0.001), inadequate food production, which increased the odds of underweight (aOR = 1.94, *p* = 0.009), and expenditure priorities focused on bills and school fees, which were also linked to higher odds of underweight (aOR = 2.46, *p* = 0.007). These associations remained significant after controlling for other factors in the model, indicating the importance of socio-economic and caregiving conditions in influencing childhood underweight status.

**Table 5:** Relationship between the contextual factors and child underweight.

Underweight	Odds Ratio	*p*-value	Confidence interval
Underweight	1		
Child’s age in months			
6–8	1.56	0.559	0.350–6.975
9–11	2.76	0.139	0.720–10.590
12–17	10.29***	<0.001	2.992–35.362
18–23	7.42***	0.002	2.096–26.259
24–35	11.58***	<0.001	3.548–37.800
36–47	6.47***	0.002	1.938–21.632
48–59	15.24***	<0.001	4.359–53.260
Disability status of care giver-able bodied	0.14***	<0.001	0.047–0.411
Adequate food production-No	1.94***	0.009	1.178–3.187
Lack of income - Yes	1.67*	0.070	0.958–2.905
Expenditure priority- bills, school fees, agro-inputs	2.46***	0.007	1.286–4.696
Access to farmers market- No	3.18*	0.096	0.816–12.395
Feeding strategy-clap hands	0.51*	0.096	0.228–1.128

Significance *** *p*<0.01, ** *p*<0.05, * *p*<0.1.

Marginally significant at the 90% level, factors such as income deprivation (aOR = 1.67, *p* = 0.070) and limited access to a farmer’s market (aOR = 3.18, *p* = 0.096) were associated with increased odds of child underweight. Conversely, certain feeding strategies, such as the “clap hands” method, were found to lower the odds of underweight in children (OR = 0.51, *p* = 0.096).

### Adaptability of nutrition intervention in relation to individual and community factors

Improving the adaptability of nutrition interventions to effectively address individual and community factors requires a comprehensive and holistic approach that prioritizes human-centered and community-driven problem-solving. This approach involves deeply understanding the unique needs, values, and circumstances of individuals and communities, while ensuring that interventions are flexible, culturally relevant, and sustainable. By focusing on the specific challenges faced by local populations and engaging them in the development and implementation of solutions, we can create more impactful, context-sensitive interventions that are better equipped to achieve lasting improvements in nutrition outcomes. Community projects thus must consider conducting baseline evaluation and consider various key elements, including age, education, access to food systems, water resources, and other contextual factors that impact nutritional outcomes.

#### Age and education

Age and education levels are critical factors when designing health education and skill-building interventions, such as culinary nutrition initiatives, particularly for under-five nutrition. The complexity of the training materials and the skills required necessitate that educational content be tailored to the literacy and educational background of caregivers to ensure effectiveness. Age-appropriate education programs can help caregivers understand the right time to introduce complementary foods and ensure these foods are culturally relevant, accessible, and nutritionally appropriate. By aligning interventions with the caregivers’ level of understanding, we can significantly enhance their ability to support the nutritional needs of their children.

#### Access to food markets

In communities where food systems are underdeveloped, food distribution and food supplements initiatives becomes a critical. Communities facing food insecurity or lack of access to nutritious food can benefit from initiatives that provide access to markets, either through mobile food markets, community-based food distribution programs, or subsidies for essential nutrients. Ensuring that families in vulnerable communities have access to nutritious, affordable food can directly address gaps in food availability and contribute to reducing under-five malnutrition.

#### Access to water

Access to clean water is foundational to many nutrition interventions, especially those involving agriculture and nutrition gardens. Agriculture-related interventions, such as nutrition gardens, depend largely on reliable access to water for irrigation and crop growth. Although this study found no significant association between nutrition gardens and malnutrition, the importance of water availability cannot be overlooked when designing interventions. Without consistent access to water, the success of agriculture produce and nutrition gardens may be limited, particularly in areas with unreliable water supply. Furthermore, water access is also critical for hygiene and sanitation, which directly impacts child nutrition outcomes.

#### Water and sanitation

Water and sanitation interventions are equally important as they directly affect the nutritional health of children. Poor sanitation and lack of clean water contribute to diarrhea-related diseases, which can increase the risk of malnutrition. Despite this study not finding a statistical significant association between the presence of a borehole and underweight in the community, it is important to recognize that limited access to clean water can increase the incidence of waterborne diseases, which can, in turn, affect nutrient absorption and overall health. Interventions aimed at improving water access and sanitation facilities are essential to reducing preventable illnesses that impair under-five nutrition.

## Discussion

The findings of this study offer valuable insights into the critical importance of adapting nutrition interventions based on empirical data gathered before implementation. By utilizing data-driven approaches, the study highlights how understanding local context and needs can enhance the effectiveness of nutrition programs. This proactive approach not only ensures that interventions are relevant and tailored to specific environments, but also helps identify potential challenges early, allowing for timely adjustments. Through this holistic perspective, the paper provides actionable insights and practical recommendations aimed at improving nutrition outcomes in diverse, underserved settings.

The strong association between age and higher odds of being underweight suggests that as children grow older, they may face increasing risks of malnutrition. These findings are in line other regional findings in Kenya, Ghana and Ethiopia (13–15). This could be due to factors such as changing nutritional needs, a shift from breastfeeding, or inadequate access to diverse, age-appropriate foods. The implication is that interventions aimed at improving child nutrition should specifically target older preschool-aged children to address this growing vulnerability. The findings also revealed that expenditure priorities play a significant role in influencing under-five malnutrition, highlighting the critical need to address financial literacy and management within communities. Families failure to prioritize spending on non-nutritional items or fail to allocate sufficient resources for essential foods, has potential to negatively impact children’s nutritional status. These findings align with similar research conducted in countries such as Ethiopia and Bangladesh, where expenditure patterns were found to be a crucial determinant of child nutrition outcomes (16, 17).

The findings also revealed that limited access to essential community resources, such as adequacy in food production and access to a farmers’ markets significantly impacts the nutritional status of under-five children. Access to adequate, diverse and nutritious food is crucial for a child’s growth and development, and the absence of these resources increases the risk of malnutrition. This insight emphasizes the importance of developing accessible, affordable, and diverse food markets and food systems within these communities. The increased odds of underweight among children in households with inadequate food production suggests that food insecurity is a critical factor contributing to child malnutrition. Families with limited access to sufficient food may struggle to meet the dietary requirements of their children, leading to underweight (18). These findings are consistent with research indicating that areas with limited access to fresh, healthy food—often referred to as “food deserts”—face higher rates of malnutrition (19, 20). In vulnerable communities, these challenges are even more pronounced, emphasizing the urgent need to improve food availability and diversity to ensure better nutrition for young children.

The finding that disability/chronic illness status of caregivers is associated with significantly lower odds of child underweight highlights the crucial role caregivers play in ensuring the nutritional well-being of children. Disability or chronic illness in caregivers often leads to reduced caregiving capacity, financial strain including difficulty in accessing food, healthcare, and essential resources. These findings align with existing research that emphasizes the profound impact of a primary caregiver’s disability and chronic ill-health on overall household welfare, including the nutritional status of children under five (21, 22). This highlights the critical need for targeted interventions that address both the caregiver’s health and the nutritional needs of vulnerable children. Feeding strategies were identified as key factors contributing to poorer nutritional outcomes in under-five children. These findings highlight the importance of prioritizing health education as essential components for improving child nutrition. Effective interventions in these areas can equip caregivers with the knowledge and skills needed to prepare nutritious meals that support children’s growth and development (23–25). The study’s results align with existing research, which suggests that context-specific interventions, informed by evidence-based data, are more likely to yield positive and sustainable impacts on child nutrition (10, 26, 27).

The study was limited to cross-sectional survey data, which provides a snapshot of factors at a single point in time and cannot establish causality. While it offers valuable insights into the contextual drivers of under-five malnutrition in a vulnerable mining community, it may not fully capture the dynamic nature of these factors or how they evolve over time. Despite these limitations, the study assessed a relatively underexplored community with a sufficiently large sample size, providing a meaningful basis for intervention planning. Future research could build on this by employing longitudinal designs to better understand the long-term effects of nutrition interventions. Additionally, exploring the economic and cost-effectiveness of these interventions across different regions could provide valuable data to inform scalable and sustainable solutions.

## Conclusion

The findings highlight the critical importance of considering contextual factors when designing nutrition interventions. A community- and human-centered approach must be applied prior to the implementation of any intervention to ensure optimal impact. Factors such as age, education, access to food markets, and availability of water and sanitation must be carefully assessed in the intervention planning process to ensure that the solutions are not only effective but also sustainable and culturally appropriate. Understanding these contextual elements allows for the design of interventions that are better aligned with the community’s specific needs, resources, and challenges. Tailoring interventions to these unique conditions increases their relevance and effectiveness, leading to improved outcomes for child nutrition. By addressing the broader social, economic, and environmental determinants of health, it is possible to create long-lasting, positive changes in the health and well-being of vulnerable populations.

## Data Availability

The raw data supporting the conclusions of this article will be made available by the authors, without undue reservation.
